# Association of *RET* codon 691 polymorphism in radiation-induced human thyroid tumours with C-cell hyperplasia in peritumoural tissue

**DOI:** 10.1038/sj.bjc.6600371

**Published:** 2002-06-17

**Authors:** A Bounacer, J A Du Villard, R Wicker, B Caillou, M Schlumberger, A Sarasin, H G Suárez

**Affiliations:** Laboratoire d'Instabilité Génétique et Cancer (UPR 2169), Institut de Recherches sur le Cancer, C.N.R.S. - IFR 89, B.P. n^o^ 8, 94801 Villejuif Cedex, France; Service de Pathologie A Institut Gustave-Roussy, 94805 Villejuif Cedex, France; Service de Médecine Nucléaire, Institut Gustave-Roussy, 94805 Villejuif Cedex, France

**Keywords:** C-cell hyperplasia, thyroid tumours, radiation, polymorphism, *RET*, *RET/PTC*

## Abstract

The *RET* proto-oncogene encodes a protein structurally related to transmembrane receptors with an intracellular tyrosine kinase domain. In human thyroid gland, the *RET* proto-oncogene is normally expressed in parafollicular C-cells. Thyroid C-cell hyperplasia is associated with inherited medullary thyroid carcinomas and is considered as a pre-neoplastic stage of C-cells disease. It has also been observed in thyroid tissues adjacent to follicular and papillary carcinomas. In order to study the relationship between a misfunctioning of the *RET* proto-oncogene and the presence of C-cell hyperplasia, we compared a series of thyroid glands presenting sporadic or radiation-associated tumours, as well as samples of unrelated normal thyroid tissues, for alteration in exons 10 and 11 of the gene and for the presence or absence of C-cell hyperplasia. Here we report a significantly higher frequency of C-cell hyperplasia present in peritumoural thyroid tissues of radiation-induced epithelial thyroid tumours, than in peritumoural of sporadic thyroid tumours or in control normal thyroid tissues (*P*=0.001). A G691S *RET* polymorphism was present with a higher frequency in radiation-induced epithelial thyroid tumours (55%) than in sporadic tumours (20%) and in control normal thyroid tissues (15%). Interestingly, this polymorphism was associated in the majority (88%) of radiation-induced tumours with a C-cell hyperplasia in the peritumoural tissues. Several explanations for this association are discussed.

*British Journal of Cancer* (2002) **86**, 1929–1936. doi:10.1038/sj.bjc.6600371 www.bjcancer.com

© 2002 Cancer Research UK

## 

Since 1950, when the first epidemiological study relating external beam radiation exposure and thyroid cancer was published (Duffy and Fitzgerald, 1950), an increased incidence of this type of tumour has been observed in populations including atomic bomb survivors (Thompson *et al*, 1994), inhabitants of regions affected by a thermonuclear test (Conard, 1984) and patients with a history of external radiation for benign or malignant conditions (Shore, 1992). Radiation-associated thyroid tumours were also observed in children contaminated in Ukraine and Belarus as a consequence of the Chernobyl accident (Kazakov *et al*, 1992).

Radiation-associated thyroid tumours are the most frequent radiation-induced tumours in man and the increase in the relative risk of developing a thyroid tumour following a radiation dose of 1 Gy to the gland during childhood, is equal to 7.7 (Ron *et al*, 1995). Studies concerning the research of genetic alterations in radiation-induced epithelial thyroid tumours, have concerned the *RAS*, *GSP*, *RET*, *TRK*, and *P53* genes (for review Suarez, 1998). These data showed a crucial role for *RET* activating rearrangements in the initiation and/or the development of the radiation-associated epithelial thyroid tumourigenic process (Suarez, 1998).

The *RET* proto-oncogene located on chromosome 10q11.2 encodes a protein structurally related to transmembrane receptors with an intracellular tyrosine kinase domain (Takahashi *et al*, 1985; Takahashi and Cooper, 1987). The ligands for RET have been recently identified as neurotrophic factors of the glial-cell-line derived neurotrophic factor (GDNF) family, including GDNF, neurturin, artemin, and perseptin (reviewed in Airaksinen *et al*, 1999; Baloh *et al*, 2000). The gene is expressed in a variety of neuronal cell lineages as well as in the kidney and enteric nervous system (Pachnis *et al*, 1993). In the normal human thyroid gland, the *RET* proto-oncogene is normally expressed in parafollicular C-cells, suggesting its involvement in the growth regulation of these cells (Fabien *et al*, 1994). The identification of germline point mutations in different domains of the *RET* proto-oncogene in inherited human diseases, namely Multiple Endocrine Neoplasia type 2A and 2B (MEN2A and MEN2B), familial or sporadic medullary thyroid carcinoma (MTC) and Hirschsprung's disease (Donis-Keller *et al*, 1993; Mulligan *et al*, 1993; Edery *et al*, 1994; Hofstra *et al*, 1994; for review Eng, 1999), confirms that this gene plays a critical role in the differentiation and growth of specific cell lineages of neural crest origin (i.e. thyroid C-cells).

Thyroid C-cell hyperplasia (CCH) was first described in the early 1970's as a lesion associated with familial MTC and MEN 2A and 2B (Wolfe *et al*, 1973; DeLellis and Wolfe, 1981; LiVolsi, 1997), and is considered as a pre-neoplastic stage of C-cell disease. CCH was also found to be associated with several other conditions. In fact, CCH was recognised in some patients with Hashimoto thyroiditis (Libbey *et al*, 1989) as well as in some other patients with chronic lymphocytic thyroiditis not within the context of MTC or MEN (Guyetant *et al*, 1994). In addition, CCH was also observed for the first time by Albores-Saavedra *et al* (1988), in thyroid tissue adjacent to follicular and papillary neoplasms.

In order to look for an eventual relationship between the presence of C-cell hyperplasia in normal thyroid tissues surrounding epithelial thyroid tumours and a possible misfunctioning of the *RET* proto-oncogene, we analysed a series of thyroid glands presenting sporadic or radiation-associated tumours as well as samples of unrelated normal thyroid tissue.

## MATERIALS AND METHODS

Tumoural thyroid tissues were collected at the Gustave Roussy Institute (Villejuif, France) and were histologically classified according to the WHO classification (Hedinger *et al*, 1988). Normal unrelated thyroid tissues were collected at the Hospital de Clinicas of Buenos Aires (Argentina) by Dr J Garcia. A total of 29 thyroid tumours obtained from patients with a history of external irradiation for benign or malignant conditions were examined: 14 follicular adenomas, 11 papillary carcinomas (PTC) and 4 widely invasive follicular carcinomas (WIFC) (Table 1

**Table 1:**
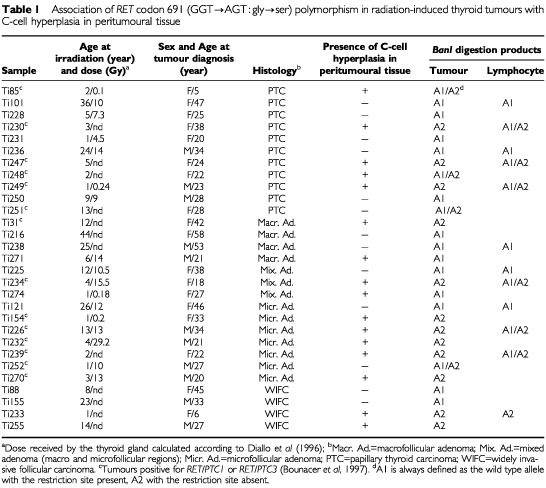
Association of *RET* codon 691 (GGT→AGT : gly→ser) polymorphism in radiation-induced thyroid tumours with C-cell hyperplasia in peritumoural tissue

). As controls we studied 29 human thyroid tumours collected from patients without any history of radiation (15 follicular adenomas, 12 PTC and 2 WIFC) (Table 2

**Table 2:**
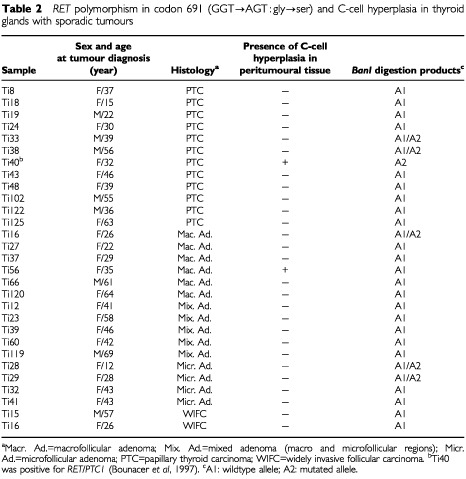
*RET* polymorphism in codon 691 (GGT→AGT : gly→ser) and C-cell hyperplasia in thyroid glands with sporadic tumours

) and 20 samples of unrelated normal thyroid tissue.

**Figure 1:**
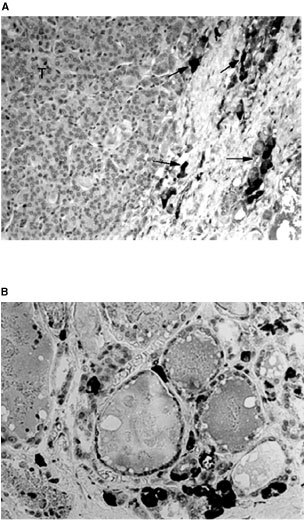
(**A**) Presence of C-cell hyperplasia in the peritumoural tissue of a radiation-induced thyroid tumour (T) of patient Ti226 (×100 magnification). The arrows indicate calcitonin positive C-cells. (**B**) The same C-cell hyperplasia seen with ×250 magnification. The calcitonin C-cells was detected by immunohistochemistry using a polyclonal anti-calcitonin antibody.

The presence of a C-cell hyperplasia (CCH) was investigated in paraffin embedded tissue sections, using an immunohistochemical technique previously described (Guyetant *et al*, 1999). The calcitonin polyclonal antibody used was from DAKO (A576). The test was performed with a streptavidin-Biotin-Peroxydase kit (LSAB-Dako-K675), after treatment with diaminobenzidine. The nuclei were stained with Mayer's Haematoxylin. The slides showing a CCH in thyroid tissue adjacent to follicular cell radio-induced tumours were carefully examined. One example of CCH in peritumoural tissue of a patient with a radiation-induced thyroid tumour is shown in Figure 1

. A C-cell hyperplasia diagnosis was made when at least three low-power fields (×100 magnification) containing more than 50 calcitonin-immunostained C-cells were observed (Guyetant *et al*, 1994).

Genomic DNA was extracted from frozen and/or paraffin embedded thyroid tissue samples, as described by Suarez *et al* (1990, 1991). Amplification of exons 10 and 11 of *RET* gene, was carried out with 250 ng genomic DNA, 250 nmol of each primer, 200 nmol dNTPs, Taq polymerase buffer (Perkin Elmer), 1.5 mmol MgCl_2_ and 2 U Taq DNA polymerase (Perkin Elmer Cetus). The following temperature cycling conditions were used: one cycle 3 min at 94°C and 2 min at 68°C (exon 10) or 60°C (exon 11), followed by 35 cycles of 30 s at 94°C, 30 s at 68°C (exon 10) or 60°C (exon 11) and 1 min at 72°C. At the end of the 35 cycles, the PCR products were extended for 10 min at 72°C. Two pairs of primers were used to amplify exons 10 and 11 of the *RET* gene. These primers were: exon 10: (sense) 5′-gcgccccaggaggctgagtg-3′ and (anti-sense) 5′-cgtggtggtcccggccgcc-3′; exon11: (sense) 5′-gcatacgcagcctgtaccc-3′ and (anti-sense) 5′-aagcttgaaggcatcccggccgcc-3′.

Direct sequence analysis of the amplified DNA fragments was carried out by the dideoxy-nucleotide method with [γ^33^P] ATP, using the double strand DNA cycle sequencing system kit (BRL, Life Technologies) and the same primers as those employed for the amplification, following the manufacturer's conditions. The reaction mixtures were then resolved on standard 8% acrylamide sequencing gels. Following electrophoresis, gels were dried and autoradiographed with X-ray film overnight.

**Figure 3:**
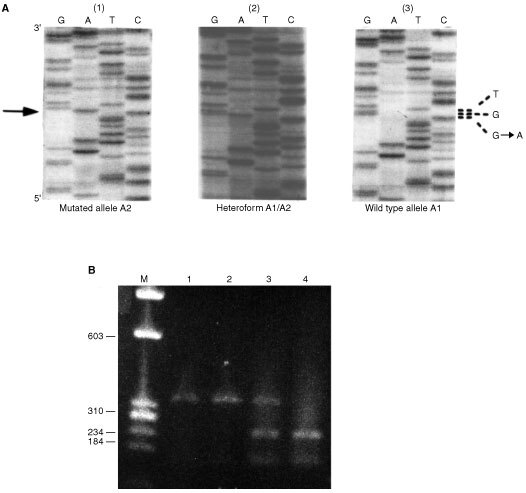
Example of G691 SNP in patient Ti226. (**A**) Direct sequence of exon 11 amplified DNA: (1) radiation-induced thyroid tumour showing mutated allele A2; (2) lymphocytic DNA showing heterozygote form (A1+A2); (3) normal thyroid sample showing wild type allele A1. (**B**) *Ban*I restriction enzyme digestion of exon 11 amplified DNA: (1) non-digested; (2) radiation associated tumour (one band: allele A2); (3) lymphocytic DNA (three bands: alleles A1+A2) and (4) normal thyroid sample (two bands: allele A1). In **A** and **B** the material studied after PCR is the same. In **B** M: marker Ø×174/*Hae*III digested DNA; 2% agarose gell stained with ethidium bromide. In **A** the arrow indicates the location of the transition G to A.

To look for the presence of an eventual polymorphism in codon 691 *RET* (see below) by restriction enzyme digestion, 20 μl of purified exon 11 amplification product, was digested with 20 U of *Ban*I (Biolabs) at 37°C all overnight for a complete digestion. After incubation the samples were separated by electrophoresis in a 2% agarose gel (see Figure 3

). Indeed, when a GGT→AGT sequence variant at codon 691 is present, there is loss of a *Ban*I restriction site and only one fragment of 408 bp (mutated allele A2) is detected instead of normally two fragments of 185 and 223 bp (wild type allele A1).

Statistical analysis was made using the Chi-square test to determine whether the associations between radiation-induced thyroid tumours with follicular phenotypes and CCH; and between G691S *RET* sequence variant and CCH, were significant.

## RESULTS

The population of patients receiving therapeutic radiation in infancy consisted of 29 subjects (18 women, 11 men; sex ratio F/M: 1.63), ranging in age at diagnosis from 5 to 58 years, with a mean age of 29.8 years. Only two patients were over 50 years of age (Table 1). The immunohistochemical study showed that 16 patients (55%), 10 women and 6 men, had C-cell hyperplasia (CCH) in the non-neoplastic peritumoural thyroid tissue. The tumours of these 16 irradiated patients were classified as follicular adenoma (Ad) in nine of 14 (64%), papillary carcinoma (PTC) in five of 11 (45%), and widely invasive follicular carcinoma (WIFC) in two of 4 (50%) (Table 1). As control, a total of 29 sporadic thyroid tumours obtained from patients without any history of radiation (sex ratio F/M: 2.6; average age at diagnosis: 40.4 years) and 20 unrelated normal thyroid tissues were screened for CCH. The C-cell hyperplasia was present in just 7% (2/29) of the thyroid glands presenting a sporadic tumour (1/15 Ad: 6.7%, 1/12 PTC: 8.4%, and 0/2 WIFC) (Table 2), and 10% (2/20) of the unrelated normal thyroid tissues (data not shown).

The C-cell hyperplasia was observed in normal tissue surrounding tumours from all patients who had received external radiation before the age of 15, with an average of 4.6 years (16/16; Table 1). There was no relationship between the dose of radiation to the thyroid and the presence of CCH (Table 1). One example of CCH, as defined in Materials and Methods, in peritumoural tissue of a patient with a radiation-induced thyroid tumour is shown in Figure 1. As expected, all thyroid tumours, adenomas, follicular carcinomas, and papillary carcinomas were calcitonin negative.

We looked then for the eventual presence of *RET* genetic alterations in our radiation-associated and sporadic tumours as well as in samples of unrelated normal thyroid tissue. We began our study investigating the presence or absence of point mutations in exons 10 and 11 of the gene. After PCR the amplified DNAs were directly sequenced. No mutations were detected in exon 10. However, a sequence variant in codon 691 of exon 11, changing a G to an A (GGT→AGT: gly→ser) and giving rise to a single nucleotide polymorphism (SNP) already described in the literature (Bugalho *et al*, 1994, Ceccherini *et al*, 1994; Gardner *et al*, 1994), was observed in 55% of the thyroid radiation-associated tumours (16/29). The frequency of this SNP, which eliminates a *Ban*I restriction site, was similar in radiation-associated follicular adenomas and carcinomas (8/14 Ad: 57%, 6/11 PTC: 54.5%, and 2/4 WIFC: 50%; Table 3

**Table 3:**
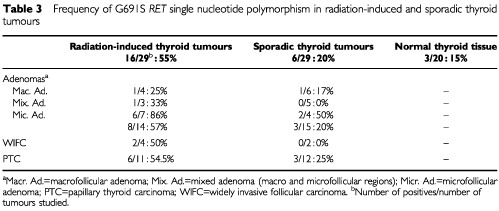
Frequency of G691S *RET* single nucleotide polymorphism in radiation-induced and sporadic thyroid tumours

). Among the adenomas, a higher frequency of SNP was observed in microfollicular tumours (6/7: 86%), whereas the frequency was similar in the follicular or papillary carcinomas (Table 3). This 691 *RET* sequence variant was also detected in 20% of sporadic tumours (6/29) and 15% of the control normal thyroid tissues (3/20) (Table 3). Again the highest frequency of SNP among the sporadic tumours was observed in the microfollicular adenomas (2/4: 50%).

**Figure 2:**
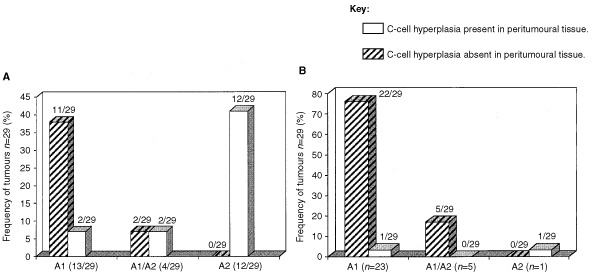
Frequency of radiation-induced (**A**) and sporadic (**B**) thyroid tumours (*n*=29) presenting a wild type allele A1, or mutated allele A2, or both (A1+A2) in the presence or absence of C-cell hyperplasia in peritumoural tissue. C-cell hyperplasia was associated with 55% (16/29) of the tumours in (**A**) and with only 7% (2/29) of the tumours in (**B**).

With the aim of determining a relationship between C-cell hyperplasia and the G691S *RET* SNP, we looked in the same thyroid sample for the polymorphism in the tumoural tissue and for the CCH in the surrounding peritumoural tissue. Our results showed that firstly, the majority of the radiation-induced tumours associated with a CCH (14/16: 88%), presented the polymorphism and interestingly, in 75% of the cases (12/16) only the mutated allele A2 was detected. Secondly, in the absence of CCH in peritumoural tissue only 14% (2/13) of the radiation-induced tumours presented a 691 *RET* sequence variant in heterozygote form (A1/A2) (Table 1 and Figure 2

).

In the sporadic thyroid tumours, the C-cell hyperplasia was observed in peritumoural tissue of only two of the 29 samples (7%) which one of them presented only a mutated allele A2 (Table 2 and Figure 2). No G691S *RET* sequence variant was detected in the DNA prepared from two of 20 samples of unrelated normal thyroid tissues presenting a CCH. Three of the 18 samples remaining were scored for the G691S *RET* SNP at heterozygote form (A1/A2) (data not shown).

The blood samples were collected from 12 of our patients with radiation-induced thyroid tumours, and the DNAs extracted from the lymphocytes were screened for the G691S *RET* sequence variant. Among them, seven samples were from patients with tumours associated with a C-cell hyperplasia in peritumoural tissue (Ti 226, 230, 233, 234, 239, 247 and 249 in Table 1). Six of them were heterozygous (A1/A2) for the G691S SNP and interestingly, in all of the cases the wild type allele A1 was lost in the tumours (loss of heterozygosity?). The only exception was the case Ti 233 in which the tumour as well as the lymphocytes showed only the mutated allele A2. The DNA of lymphocytes of the other five patients whose radiation-induced tumours were not associated with a CCH presented as in the tumours, only a 691 codon wild type sequence (Ti 101, 155, 225, 236 and 238 in Table 1). All the radiation-associated tumours presenting a G691S *RET* SNP, with the exception of samples Ti 233 and 255, were positive for a *RET/PTC1* or *RET/PTC3* rearrangement. This was also the case for the sporadic tumoural sample Ti 40 (Bounacer *et al*, 1997; Tables 1 and 2).

Examples of the *RET* G691S *RET* sequence variant studied by sequence or restriction enzyme digestion, are given in Figure 3.

## DISCUSSION

Our results show a significantly higher frequency of C-cell hyperplasia in peritumoural thyroid tissues of radiation-induced epithelial thyroid tumours (55%), than in peritumoural tissues of sporadic thyroid tumours (7%) or in control normal thyroid tissues (10%) (*P*=0.0001, Chi-square test). The presence of CCH in the non-neoplastic tissue adjacent to follicular cell tumours was previously reported with a frequency of 35% by Albores-Saavedra *et al* (1988). However, the authors defined CCH when at least 50 C-cells were observed in only one lower power field (×100 magnification) rather than at least three fields according to our criteria, and probably some of their positive cases will be taken by us as a negative.

Several conditions such as hyperparathyroidism, hypercalcemia, infancy and chronic lymphatic thyroiditis (Wolfe *et al*, 1975a,b; Gibson *et al*, 1980; DeLellis, 1992; Tomita and Millard, 1992), are now admittedly associated with CCH, whereas others, such as age and sex, are still subject to controversy (Gibson *et al*, 1982; O'Toole *et al*, 1985; Albores-Saavedra *et al*, 1988; Scopsi *et al*, 1991; Guyetant *et al*, 1997; Harach, 1997). In our patients with a history of external radiation, no correlation has been seen in the sex ratio between the CCH positive and CCH negative groups (1.66 and 1.6 respectively). Moreover, we did not observe any significant difference (using Mann–Witney analysis) according to age at diagnosis between these two groups (mean age: 26.6 years in CCH positive group (excluding the youngest patients Ti85 and Ti233) and 37 years in CCH negative group). However, all of the patients showing the CCH in normal tissue surrounding tumours had received external radiation in infancy before the age of 15 (with an average of 4.6 years) and their tumours occurred with an average of 20 years. According to the fact that up to date there is no data reporting that medullary thyroid carcinomas, in which CCH is considered as a pre-neoplastic stage, are associated with radiation, we can postulate an indirect role of irradiation in the occurrence of CCH. In fact, we have shown the presence of a sequence variant (GGT→AGT: gly→ser), in codon 691 of exon 11 of the *RET* gene, giving rise to a polymorphism, in 55% of radiation-associated thyroid tumours. This polymorphism was present in the majority (88%) of these radiation-induced tumours associated with a CCH in peritumoural tissues. Interestingly, in 75% of these samples only the mutated allele A2 was detected. In the absence of CCH, the polymorphism was observed in a minority of the radiation-induced tumours in a heterozygous form (A1/A2).

In sporadic epithelial thyroid tumours and in normal thyroid tissues, the frequency of this polymorphism was similar (15 to 20%) and significantly lower than in radiation-associated tumours (*P*=0.0032, Chi-square test). Moreover, the C-cell hyperplasia was observed in peritumoural tissue of only two of the 29 sporadic thyroid tumours studied and just one of them presented a serine residue (allele A2) on the codon 691 of the RET protein. In all the other normal or tumoural sporadic thyroid tissues studied for which the CCH was not observed, the sequence of the codon 691 *RET* was in wild type (majority of cases) or in heterozygote form (A1/A2).

Our data indicate a correlation between the presence of a C-cell hyperplasia in peritumoural irradiated thyroid tissue and the presence of the mutated sequence in codon 691 of the RET protein (allele A2) in neighbouring epithelial thyroid tumours. The molecular bases of this relationship are actually unknown. The possibility of the existence of some functional interconnections between follicular and parafollicular C-cells, has been recently evoked. For instance, Matias-Guiu (1999) and Volante *et al* (1999) suggested that the microenvironment provided by MTC cells may have the capacity to stimulate the proliferation of follicular cells, giving rise to hyperplastic and/or adenomatous follicles which, sometimes, may evolve in these conditions to a fully neoplastic phenotype. The opposite situation has also been described: the presence of CCH in thyroid glands with Hashimoto's thyroiditis or adjacent to benign or malignant epithelial tumours (Albores-Saavedra *et al*, 1988; Libbey *et al*, 1989; and our present data). Furthermore, it has been also recently observed by Cosci *et al* (2000) that the allele variants of *RET* G691S in exon 11 are significantly more frequent in patients with sporadic MTC than in the general population. Moreover, it has been reported that a neutral germline sequence variance S836S *RET* may somehow predispose to sporadic MTC, especially those that harbour somatic M918T mutation (Gimm *et al*, 1999). A highly significant association of RET polymorphisms, specifically the variant A45A, with Hirschsprung disease has also been observed (Borrego *et al*, 1999, 2000; Fitze *et al*, 1999). Taking into account these and our present data, we suggest that the higher frequency of CCH observed in the irradiated thyroid glands of the patients bearing in their tumours a G691S *RET* SNP, may be an effect of the RET allele (or haplotype) on which the sequence variant has occurred.

The precise mechanism by which G691S affect the function of RET protein is unknown and open to speculation. It has been shown that polymorphic sequence variants can lead to production of different amounts of mRNA (Leviev *et al*, 1997). It may be suggested that the GGT→AGT polymorphism causes the creation of a cryptic splice donor, splice acceptor or splice enhancer, therefore leading to an altered protein that may contribute to the development of C-cell hyperplasia. Similar mechanisms have been previously hypothesised in the cases of polymorphisms associated with sporadic MTC and Hirschsprung disease (Borrego *et al*, 1999; Fitze *et al*, 1999; Gimm *et al*, 1999). Unfortunately, RNA from our radio-induced thyroid tumours was not available to test this hypothesis. It can be also postulated when an amino acid is altered for example G691S, depending on the genotype, could subtly alter the function of the RET protein if located in a critical domain. If as a consequence of the radiation received by the thyroid, a pre-existing heterozygous G691S SNP becomes homozygous, the RET protein may be sufficiently affected to overcome a threshold of activation and, alone or interacting with other molecules, induce by still unknown mechanisms an accelerated growth of C-cells (see below). This may explain the fact that the growth of C-cells was not affected in 15% of our normal thyroid tissues and 20% of the sporadic tumours, by the presence of an A1/A2 heterozygous form. This hypothesis may be supported by data obtained studying the DNA of lymphocytes of some of our patients who presented simultaneously, in their peritumoural thyroid tissues a CCH, and in their radiation-associated tumours only the mutated allele A2. Indeed, the majority of these lymphocytic DNAs (6/7 samples) showed a heterozygous G691S *RET* variant sequence (A1/A2), suggesting a probable loss of the wild type allele A1 in the tumour samples. Unfortunately, lymphocytic material was not available for all the studied cases; we can speculate a probable similar situation for the cases in which the radiation-associated tumours presented only the mutated allele A2 in association with a CCH in peritumoural tissues.

Interestingly, all our radiation-induced thyroid tumours (except Ti233 and Ti255) presenting the mutated allele A2 and showing a CCH in peritumoural tissues are positive for *RET/PTC* rearrangements (Table 1 and Bounacer *et al*, 1997). This association between *RET/PTC* and the allele A2 may contribute to a CCH observed in peritumoural tissues of these tumours. The hypothesis that an eventual stimulation of RET expression in tumoural follicular cells may give rise to the development of a CCH in their environment, can be supported by recent data from Bunone *et al* (2000). Indeed these authors showed that there is RET expression in thyroid benign or malignant tumoural follicular cells and in these cells the *RET* promoter is always active after *RET/PTC* rearrangement. They reported also that a functional proto-*RET* receptor might be expressed in epithelial thyroid carcinomas in the absence of *RET/PTC*. Finally, the authors concluded that the stimulation of RET expression may contribute to a simultaneous or alternative higher proliferation of both follicular and neighbouring parafollicular cells. In this context, we cannot exclude that the mutated G691S *RET* allele, over-represented in the epithelial radiation-associated tumours compared to controls, may lie in linkage disequilibrium with other sequences that may confer low level predisposition to or protection against anarchic growth of C-cells. Furthermore, the possibilities of an interaction of the modified RET protein with other molecules to stimulate C-cell growth must not be neglected.

Theoretically, polymorphisms represent sequence variations, which are present in the general population and confer no obvious or important deleterious effects. However, it becomes clear that some polymorphisms like the APC gene in colorectal cancer in the Ashkenazim (Laken *et al*, 1997) and the paraoxonase gene in coronary heart disease in type 2 diabetes (Ruiz *et al*, 1995) are not entirely harmless. These observations taken together with our present data argue in favour that *RET* G691S variant can constitute a factor contributing to the development of CCH in the peritumoural tissues of irradiated thyroid glands. Further efforts must be aimed to confirm a loss of the 691 *RET* wild type allele in the irradiated thyroid tumours associated with a CCH; and also to clarify by which mechanisms the microenvironment provided by these tumours positive for G691S mutated allele has the capacity to stimulate the development of CCH.

## References

[bib1] AiraksinenMSTitievskyASaarmaM1999 GDNF family neurotrophic factor signaling: four masters, one servant? Mol Cell Neurosci 13 313325 10356294 10.1006/mcne.1999.0754

[bib2] Albores-SaavedraJMonforteHNadjiMMoralesAR1988 C-cell hyperplasia in thyroid tissue adjacent to follicular cell tumors Hum Pathol 19 795799 2900208 10.1016/s0046-8177(88)80262-4

[bib3] BalohRHEnomotoHJohnsonJrEMMilbrandtJ2000 The GDNF family ligands and receptors - implications for neural development Curr Opin Neurobiol 10 103110 10679429 10.1016/s0959-4388(99)00048-3

[bib4] BorregoSRuizASaezMEGimmOGaoXLopez-AlonsoMHernandezAWrightFAAntinoloGEngC2000 RET genotypes comprising specific haplotypes of polymorphic variants predispose to isolated Hirschsprung disease J Med Genet 37 572578 10922382 10.1136/jmg.37.8.572 PMC1734658

[bib5] BorregoSSaezMERuizAGimmOLopez-AlonsoMAntinoloGEngC1999 Specific polymorphisms in the RET proto-oncogene are over-represented in patients with Hirschsprung disease and may represent loci modifying phenotypic expression J Med Genet 36 771774 10528857 10.1136/jmg.36.10.771 PMC1734238

[bib6] BounacerAWickerRCaillouBCailleuxAFSarasinASchlumbergerMSuarezHG1997 High prevalence of activating ret proto-oncogene rearrangements, in thyroid tumors from patients who had received external radiation Oncogene 15 12631273 9315093 10.1038/sj.onc.1200206

[bib7] BugalhoMJCoteGJKhoranaSSchultzPNGagelRF1994 Identification of a polymorphism in exon 11 of the RET protooncogene Hum Mol Genet 3 2263 10.1093/hmg/3.12.2263-a 7881435

[bib8] BunoneGUggeriMMondelliniPPierottiMABongarzoneI2000 RET receptor expression in thyroid follicular epithelial cell-derived tumors Cancer Res 60 28452849 10850426

[bib9] CeccheriniIHofstraRMLuoYStulpRPBaroneVStelwagenTBocciardiRNijveenHBolinoASeriMRonchettoPPasiniBBozzanoMBuysCRomeoG1994 DNA polymorphisms and conditions for SSCP analysis of the 20 exons of the ret proto-oncogene Oncogene 9 30253029 8084609

[bib10] ConardR1984 Late radiation effects in Marshall islanders exposed to fallout 28 years agoInRadiation carcinogenesis: epidemiology and biology significanceBoice JD, Fraumeni JF (eds)pp5770New York: Raven Press

[bib11] CosciBRomeiCVivaldiABotticiVRosselliRBonottiALariRPincheraA2000 RET exon 11 (G691) polymorphism is significantly more frequent in medullary thyroid carcinoma than in the general population (Abstract) J Endocrinol Invest 23 510698044

[bib12] DeLellisRAWolfeHJ1981 The pathobiology of the human calcitonin (C)-cell: a review Pathol Annu 16 2552 7036062

[bib13] DeLellisRA1992 C-cell hyperplasiaInAtlas of tumor pathology, 3rd series, fasc 5: Tumors of the thyroid glandRosai J, Carcangiu ML, DeLellis RA (eds)pp247258Washington, DC: Armed Forces Institute of Pathology

[bib14] DialloILamonAShamsaldinAGrimaudEde VathaireFChavaudraJ1996 Estimation of the radiation dose delivered to any point outside the target volume per patient treated with external beam radiotherapy Radiother Oncol 38 269271 8693110 10.1016/0167-8140(96)01713-6

[bib15] Donis-KellerHDouSChiDCarlsonKMToshimaKLairmoreTCHoweJRMoleyJFGoodfellowPWellsJrSA1993 Mutations in the RET proto-oncogene are associated with MEN 2A and FMTC Hum Mol Genet 2 851856 8103403 10.1093/hmg/2.7.851

[bib16] DuffyBJJFitzgeraldPJ1950 Cancer of the thyroid in children: A report of 28 cases J Clin Endocrinol Metab 10 12961308 14794754 10.1210/jcem-10-10-1296

[bib17] EderyPLyonnetSMulliganLMPeletADowEAbelLHolderSNihoul-FeketeCPonderBAMunnichA1994 Mutations of the RET proto-oncogene in Hirschsprung's disease Nature 367 378380 8114939 10.1038/367378a0

[bib18] EngC1999 RET proto-oncogene in the development of human cancer J Clin Oncol 17 380393 10458257 10.1200/JCO.1999.17.1.380

[bib19] FabienNPaulinCSantoroMBergerNGriecoMDuboisPMFuscoA1994 The RET proto-oncogene is expressed in normal human parafollicular thyroid cells Int J Oncol 4 623626 21566968 10.3892/ijo.4.3.623

[bib20] FitzeGSchreiberMKuhlischESchackertHKRoesnerD1999 Association of RET protooncogene codon 45 polymorphism with Hirschsprung disease Am J Hum Genet 65 14691473 10521317 10.1086/302618 PMC1288303

[bib21] GardnerEMulliganLMEngCHealeyCSKwokJBPonderMAPonderBA1994 Haplotype analysis of MEN 2 mutations Hum Mol Genet 3 17711774 7849700 10.1093/hmg/3.10.1771

[bib22] GibsonWCrokerBCoxC1980 C-cell populations in normal children and young adults Lab Invest 42 119120

[bib23] GibsonWGPengTCCrokerBP1982 Age-associated C-cell hyperplasia in the human thyroid Am J Pathol 106 388393 6175219 PMC1916228

[bib24] GimmONeubergDSMarshDJDahiaPLHoang-VuCRaueFHinzeRDralleHEngC1999 Over-representation of a germline RET sequence variant in patients with sporadic medullary thyroid carcinoma and somatic RET codon 918 mutation Oncogene 18 13691373 10022819 10.1038/sj.onc.1202418

[bib25] GuyetantSDupreFBigorgneJCFrancBDutrieux-BergerNLecomte-HouckeMPateyMCaillouBViennetGGuerinOSaint-AndreJP1999 Medullary thyroid microcarcinoma: a clinicopathologic retrospective study of 38 patients with no prior familial disease Hum Pathol 30 957963 10452509 10.1016/s0046-8177(99)90250-2

[bib26] GuyetantSRousseletMCDurigonMChappardDFrancBGuerinOSaint-AndreJP1997 Sex-related C cell hyperplasia in the normal human thyroid: a quantitative autopsy study J Clin Endocrinol Metab 82 4247 8989230 10.1210/jcem.82.1.3684

[bib27] GuyetantSWion-BarbotNRousseletMCFrancBBigorgneJCSaint-AndreJP1994 C-cell hyperplasia associated with chronic lymphocytic thyroiditis: a retrospective quantitative study of 112 cases Hum Pathol 25 514521 8200646 10.1016/0046-8177(94)90124-4

[bib28] HarachHR1997 Age, sex and C cells: what about the human thyroid follicle with acid mucin and solid cell nests? J Clin Endocrinol Metab 82 4274 10.1210/jcem.82.12.4466-1 9432519

[bib29] HedingerCWilliamsEDSobinLH1988 Histological typing of thyroid tumoursIn2nd edNo 11 of International histological classification of tumoursHedinger C, Williams ED and Sobin LH (eds)Berlin, Germany: Springer-Verlag

[bib30] HofstraRMLandsvaterRMCeccheriniIStulpRPStelwagenTLuoYPasiniBHoppenerJWvan AmstelHKRomeoGLipsCBuysC1994 A mutation in the RET proto-oncogene associated with multiple endocrine neoplasia type 2B and sporadic medullary thyroid carcinoma Nature 367 375376 7906866 10.1038/367375a0

[bib31] KazakovVSDemidchikEPAstakhovaLN1992 Thyroid cancer after Chernobyl Nature 359 21

[bib32] LakenSJPetersenGMGruberSBOddouxCOstrerHGiardielloFMHamiltonSRHampelHMarkowitzAKlimstraDJhanwarSWinawerSOffitKLuceMCKinzlerKWVogelsteinB1997 Familial colorectal cancer in Ashkenazim due to a hypermutable tract in APC Nat Genet 17 7983 9288102 10.1038/ng0997-79

[bib33] LevievINegroFJamesRW1997 Two alleles of the human paraoxonase gene produce different amounts of mRNA. An explanation for differences in serum concentrations of paraoxonase associated with the (Leu-Met54) polymorphism Arterioscler Thromb Vasc Biol 17 29352939 9409279 10.1161/01.atv.17.11.2935

[bib34] LibbeyNPNowakowskiKJTucciJR1989 C-cell hyperplasia of the thyroid in a patient with goitrous hypothyroidism and Hashimoto's thyroiditis Am J Surg Pathol 13 7177 2909199 10.1097/00000478-198901000-00011

[bib35] LiVolsiVA1997 C cell hyperplasia/neoplasia J Clin Endocrinol Metab 82 39418989229

[bib36] Matias-GuiuX1999 Mixed medullary and follicular carcinoma of the thyroid. On the search for its histogenesis Am J Pathol 155 14131418 10550294 10.1016/S0002-9440(10)65453-3 PMC1866975

[bib37] MulliganLMKwokJBHealeyCSElsdonMJEngCGardnerELoveDRMoleSEMooreJKPapiLPonderMATeleniusHTunnacliffeAPonderBAJ1993 Germ-line mutations of the RET proto-oncogene in multiple endocrine neoplasia type 2A Nature 363 458460 8099202 10.1038/363458a0

[bib38] O'TooleKFenoglio-PreiserCPushparajN1985 Endocrine changes associated with the human aging process: III. Effect of age on the number of calcitonin immunoreactive cells in the thyroid gland Hum Pathol 16 9911000 3899904 10.1016/s0046-8177(85)80276-8

[bib39] PachnisVMankooBCostantiniF1993 Expression of the c-ret proto-oncogene during mouse embryogenesis Development 119 10051017 8306871 10.1242/dev.119.4.1005

[bib40] RonELubinJHShoreREMabuchiKModanBPotternLMSchneiderABTuckerMABoiceJrJD1995 Thyroid cancer after exposure to external radiation: a pooled analysis of seven studies Radiat Res 141 259277 7871153

[bib41] RuizJBlancheHJamesRWGarinMCVaisseCCharpentierGCohenNMorabiaAPassaPFroguelP1995 Gln-Arg192 polymorphism of paraoxonase and coronary heart disease in type 2 diabetes Lancet 346 869872 7564671 10.1016/s0140-6736(95)92709-3

[bib42] ScopsiLDi PalmaSFerrariCHolstJJRehfeldJFRilkeF1991 C-cell hyperplasia accompanying thyroid diseases other than medullary carcinoma: an immunocytochemical study by means of antibodies to calcitonin and somatostatin Mod Pathol 4 297304 1676840

[bib43] ShoreRE1992 Issues and epidemiological evidence regarding radiation-induced thyroid cancer Radiat Res 131 98111 1385649

[bib44] SuarezHG1998 Genetic alterations in human epithelial thyroid tumours Clin Endocrinol (Oxf) 48 531546 9666864 10.1046/j.1365-2265.1998.00443.x

[bib45] SuarezHGdu VillardJACaillouBSchlumbergerMParmentierCMonierR1991 gsp mutations in human thyroid tumours Oncogene 6 677679 1903197

[bib46] SuarezHGdu VillardJASeverinoMCaillouBSchlumbergerMTubianaMParmentierCMonierR1990 Presence of mutations in all three ras genes in human thyroid tumors Oncogene 5 565570 2183158

[bib47] TakahashiMCooperGM1987 ret transforming gene encodes a fusion protein homologous to tyrosine kinases Mol Cell Biol 7 13781385 3037315 10.1128/mcb.7.4.1378-1385.1987 PMC365224

[bib48] TakahashiMRitzJCooperGM1985 Activation of a novel human transforming gene, ret, by DNA rearrangement Cell 42 581588 2992805 10.1016/0092-8674(85)90115-1

[bib49] ThompsonDEMabuchiKRonESodaMTokunagaMOchikuboSSugimotoSIkedaTTerasakiMIzumiSPrestonDL1994 Cancer incidence in atomic bomb survivors. Part II: Solid tumors, 1958-1987 Radiat Res 137 1767

[bib50] TomitaTMillardDM1992 C-cell hyperplasia in secondary hyperparathyroidism Histopathology 21 469474 1452130 10.1111/j.1365-2559.1992.tb00433.x

[bib51] VolanteMPapottiMRothJSaremaslaniPSpeelEJLloydRVCarneyJAHeitzPUBussolatiGKomminothP1999 Mixed medullary-follicular thyroid carcinoma. Molecular evidence for a dual origin of tumor components Am J Pathol 155 14991509 10550306 10.1016/S0002-9440(10)65465-X PMC1866972

[bib52] WolfeHJDeLellisRAScottRTTashjianAH1975a C-cell hyperplasia in chronic hypercalcemia in man (abstract) Am J Pathol 78 20A

[bib53] WolfeHJDeLellisRAVoelkelEFTashjianAH1975b Distribution of calcitonin-containing cells in the normal neonatal human thyroid gland: a correlation of morphology with peptide content J Clin Endocrinol Metab 41 10761081 1206094 10.1210/jcem-41-6-1076

[bib54] WolfeHJMelvinKECervi-SkinnerSJSaadiAAJuliarJFJacksonCETashjianAH1973 C-cell hyperplasia preceding medullary thyroid carcinoma N Engl J Med 289 437441 4587234 10.1056/NEJM197308302890901

